# Hyperbaric Oxygen Therapy in Children with Brain Injury: A Retrospective Case Series

**DOI:** 10.7150/ijms.102884

**Published:** 2025-01-01

**Authors:** Michal Hajek, Ondrej Jor, Jakub Tlapak, Dittmar Chmelar

**Affiliations:** 1 Centre of Hyperbaric Medicine, Ostrava City Hospital, Ostrava, Czech Republic.; 2 Institute of Laboratory Medicine, Institute of Microbiology, Faculty of Medicine, University of Ostrava, Ostrava, Czech Republic.; 3 Centre for Hyperbaric Medicine of Faculty of Medicine University of Ostrava and Ostrava City Hospital, Ostrava, Czech Republic.; 4 Department of Anaesthesiology, Resuscitation and Intensive Care Medicine, Faculty of Medicine, University of Ostrava, Ostrava, Czech Republic; 5 Department of Anaesthesiology and Intensive Care Medicine, University Hospital of Ostrava, Ostrava, Czech Republic.; 6 The Institute of Aviation Medicine, Prague, Czech Republic.; 7 Faculty of Biomedical Engineering, Czech Technical University in Prague, Czech Republic.

**Keywords:** Traumatic Brain Injury, Hypoxic-Ischemic Encephalopathy, Hyperbaric oxygen therapy, Glasgow Coma Scale, Glasgow Outcome Scale, Case Series, Pediatric patients

## Abstract

**Introduction and Importance:** Some experimental studies on brain injury associated with traumatic brain injury (TBI) and hypoxic-ischaemic encephalopathy (HIE) reveal a positive effect of hyperbaric oxygen therapy (HBOT). However, in clinical medicine, most of the scientific evidence available in the current literature relates only to TBI.

**Methods:** The primary objective is to empirically assess the efficacy of HBOT in mitigating the symptoms of disability associated with brain injury in children, with a view to elucidating its therapeutic potential and clinical benefits.

**Outcomes:** A total of 21 patients have been treated with HBOT. The mean age was 6±4.6 years. There were 12 cases (57%) of TBI, 8 cases (38%) of HIE and 1 case (5%) of ischaemic stroke. The mean initial Glasgow Coma Scale (GCS) at hospital admission immediately after accident was 3.3±0.9. The mean time from injury to HBOT was 5.2 ± 3.8 weeks. The mean number of HBOT exposures was 10±4.3. The mean GCS pre-HBOT was 10.7±3.7 and 12.3±3.4 (p=0.004) after post-HBOT, respectively. The mean Glasgow Outcome Scale (GOS) was 3.3±0.8 pre-HBOT, and 3.9±1.1 (p<0.001) after post-HBOT, respectively. Eighteen cases were included in response to HBOT assessment. Six cases (33%) were evaluated as large clinically significant response (CSR), 7 cases (39%) were evaluated as partial response with minimally important difference (MID). Five cases (28%) were evaluated as non-response. The results showed better response to HBOT in cases of starting HBOT up to 4 weeks (p=0.02) after the injury. There was no serious HBOT-related complication or injury.

**Conclusion:** Results of our study demonstrate both clinical and statistically significant patient response to HBOT. Our data also suggest that the earlier HBOT started after diagnosis up to 4 weeks, the more pronounced patients' response to HBOT was achieved. The provision of HBOT to pediatric patients is feasible in large regional hyperbaric centers.

## Introduction

There are only a few concise papers and comprehensive communications concerning the use of HBOT in the treatment of patients of childhood age 1-4. Physicians in pediatric disciplines, both primary and adjunct (pediatric cardiologist, pediatric surgeon and orthopedic surgeon, pediatric intensivist) may potentially encounter a situation where it will be necessary to consider the inclusion of HBOT in the treatment program.

Brain injury encompasses a broad spectrum of conditions characterized by structural or functional abnormalities in the brain (encephalopathy). Hypoxic-ischemic encephalopathy (HIE) stands as a prevalent pathological condition in critical care settings, particularly following cardiac arrest. Despite advancements, the prognosis remains grim 5. Traumatic brain injury (TBI) and stroke represent the primary causes of brain damage, with TBI being one of the foremost contributors to mortality and disability within the general population 6. Current clinical practice lacks efficacious treatments or metabolic interventions for patients experiencing chronic neurological dysfunction post-TBI or stroke. While intensive therapies and rehabilitation programs are deemed imperative for optimizing quality of life, their efficacy often falls short of complete success 5-7.

TBI exerts significant adverse societal and economic ramifications. Annually, nearly 4 million individuals in the United States sustain a TBI, with approximately half necessitating emergency department visits, 500,000 requiring hospitalizations, and 50,000 succumbing to their injuries 8. About 2% of the U.S. population (5.3 million individuals) endure long-term disabilities stemming from TBI. The combined direct and indirect annual financial burden of TBI in the United States amounts to $76.5 billion 9, 10. Despite these considerable physical and financial tolls, therapeutic advancements for TBI have been scant since the 1990s, with clinical outcomes showing no improvement. Over the past 15 years, at least 25 clinical trials investigating therapeutics for TBI have proven unsuccessful 11-13.

Numerous treatments administered immediately following TBI aim to modify acute pathophysiology. However, secondary injury often ensues following the primary mechanical insult to the brain. This secondary injury typically arises from ischemia induced by reduced cerebral blood flow (CBF) 13, 14.

Hyperbaric oxygen therapy (HBOT) has been proposed to mitigate oxidative stress, inflammation, and neural apoptosis, thereby enhancing functional recovery post-stroke 15. Studies suggest that HBOT in rats afflicted with ischemic stroke promotes trophic factor expression, neurogenesis, and mobilization of bone marrow stem cells to the ischemic region, potentially facilitating cell repair 16. Notably, improvements in cognition, executive functions, physical abilities, gait, sleep, and quality of life in stroke patients persisted for up to three months post-treatment 17. In patients with mild TBI, HBOT has shown to aid in recovery during the rehabilitation phase 18.

Mounting evidence indicates that HBOT can induce neuroplasticity and enhance cognitive function in individuals with chronic neurocognitive impairment stemming from TBI, stroke, and anoxic brain damage 19-22. These changes are associated with the promotion of cerebral angiogenesis, increased CBF, and enhancements in cerebral white and gray microstructures 23, 24 HBOT has been noted to exert neuroprotective effects by elevating anti-apoptotic proteins, reducing DNA damage in cerebral ischemic cells, mitigating cerebral necrosis and edema, lowering mortality rates, minimizing secondary brain damage, and bolstering the integrity of the blood-brain barrier 7.

## Material and Methods

This work is a retrospective analysis of the case series of patients with diagnosed brain injury of traumatic and non-traumatic etiology treated in the Centre of Hyperbaric Medicine Ostrava for five years (period 2019-2023). The primary objective is to empirically assess the efficacy of HBOT in mitigating the symptoms of disability associated with brain injury in children, with a view to elucidating its therapeutic potential and clinical benefits.

### Patients

Patients with brain injury have been primarily treated at various hospitals and pediatric departments located in many regions such as the Moravian-Silesian, Olomoucký, South Moravian, Královehradecký Region as well as City of Prague, Czech Republic. Brain injury of both traumatic and non-traumatic etiology was involved, in particular patients with TBI and HIE after cardiopulmonary resuscitation (CPR). After a referral phone call regarding the suitability of the indication and the occurrence or exclusion of contraindications for HBOT in a given patient between the treating specialist and the physician of the hyperbaric center, logistics were subsequently agreed. Subsequently, the management of the children's department was approached to discuss treatment options and provision of bed capacity for hospitalization of patient and ensuring availability of nurses, who routinely provide staffing and care inside the hyperbaric chamber for children under 10 years of age. Based on clinical status and low initial GCS values, our patients had been diagnosed with severe brain injuries.

In our workplace, we normally allow the treatment of pediatric patients with this type of brain injury, however, for safety reasons, in a delayed mode (subacute to early chronic stage), rather than in the acute stage. The initiation of HBOT was then scheduled for a minimum of 2-3 weeks after the injury, after general stabilization of the condition of patient. All suggested eligible patients without any contraindications and restrictions were consecutively admitted to our center. The following exclusion criteria were applied: Carbon Monoxide intoxication, oncological or haemato-oncological disease, ongoing acute infection, severe decompensation of organ functions or organ failure, condition requiring artificial pulmonary ventilation. An analysis of the medical records of all patients was performed and demographic data, history, associated diseases, severity of injury, neurological functional impairment, surgical procedures, Glasgow Coma Scale (GCS) and Glasgow Outcome Scale (GOS) pre- and post-HBOT, time from injury to HBOT, number of HBOT exposures, medication used during HBOT and other parameters were evaluated.

### HBOT treatment

HBOT therapy was administered in a multiplace hyperbaric chamber produced at the Vítkovice Steel company in Czechia, renovated by Haux-Life-Support in Germany. Prior to commencing treatment, clinical evaluation by an ear, nose, and throat (ENT) specialist was conducted, assessing the patients' ability to compensate for pressure changes in the middle ear cavities. In cases of impaired consciousness, bilateral paracentesis or insertion of ventilation tubes was performed. Parents of the children undergoing HBOT were informed about the treatment and provided informed consent. All patients underwent initial evaluation by a pediatrician and a physician from the HBOT unit. Patients were accompanied by healthcare professionals, typically a pediatric nurse, pediatrician, or hyperbaric nurse throughout the stay in the hyperbaric chamber. It was the responsibility of healthcare professionals, particularly nurses, to monitor patients and promptly inform a hyperbaric physician in case of serious adverse events or intolerance to HBOT.

The HBOT was applied at 200 kPa, or 2.0 absolute atmosphere (ATA) once a day. Oxygen breathing lasted 75-80 minutes, with two 5 minutes air breaks included. The compression and decompression rates ranged from 6-10 kpa/min. Each patient had a minimum number of 10-15 exposures scheduled at the start of treatment. The total number of exposures then depended on the course and the tolerance of HBOT, the development of the general condition, changes of the neurological functions and the occurrence of adverse effects.

### Discomfort and adverse effects

All adverse effects occurring during HBOT especially those requiring treatment or therapeutic intervention, such as medication, modification of treatment regimen, interruption or discontinuation of treatment were evaluated and reported.

### Assessment of consciousness and functional prognostic status

GCS scores varied from 3 to 15 points, with 15 points indicating clear consciousness, 12 to 14 points signifying mild disturbance of consciousness, 9 to 11 points indicating moderate disturbance of consciousness, and 8 points indicating coma. A GOS score of 5 points indicated successful recovery with a return to a normal state, albeit with minor impairments; 4 points indicated moderate disability, allowing independent living and work with precautions; 3 points indicated consciousness with severe disability necessitating daily life assistance; 2 points indicated a vegetative state with minimal responsiveness; and 1 point indicated death.

The initial GCS measurement was performed during the admission to the hospital immediately after the injury. GCS and GOS in relation to HBOT therapy were assessed by physician twice for each patient: during the admission of the patient to hospital in the pediatric ward or by hyperbaric physician during initial (pre-HBOT) or final HBOT session (post-HBOT). GCS and GOS measurements were performed before the administration of sedatives.

The initial GCS assessment was therefore logically determined on average more than 5 weeks before the pre-HBOT GCS assessment. The children were treated in a comprehensive manner including surgical methods during this period and were hospitalised in the ICU and later in the standard ward of paediatric department. This is the main reason why the values of initial GCS and the pre-HBOT GCS are so different. It is therefore, of course, a result of natural treatment process.

### Response to HBOT evaluation

Response to HBOT was assessed as a large clinically significant response (CSR) when there was an improvement of 2 points or more in GOS score or 1 point in GOS score and concurrently 3 or more in GCS. Minimally important difference (MID) to treatment was assessed as an improvement of 1 point on the GOS score and/or at least 1 point on GCS score. The “overall response rate” is the summation of the CSR and MID. “No response” is used when the symptom does not show any improvement after therapy.

### Method of processing and analysis of collected data

Data on patients were obtained by detailed analysis of all patient medical charts and by inclusion into the Microsoft Excel program (Microsoft Office Standard 2013). The above was done by one member of the team. None of the other members of the team participated in this activity. On the contrary, another member of the team was responsible for processing the supplied data and files into tables, statistical processing and writing the first draft of manuscript.

### Statistical assessment

Mean values, standard deviation (SD), median and quartiles values were used to describe demographic datasets, number of HBOT exposures and treatment outcomes. Differences in assessment of GCS and GOS scores before and after HBOT therapy were assessed by the Wilcoxon Test. Response to HBOT therapy in relation to time from injury to HBOT was assessed by the Pearson Chi-Square Test (p value less than 0.05 was considered statistically significant).

### Ethical considerations

This work used retrospective methods and therefore ethical approval was waived. There is no requirement in the legislation of the Czech Republic for assessment and approval of research projects that do not meet the definition of a clinical trial of medicinal product or a clinical trial of medical device. The issue of Ethics Committees is regulated by the Act No. 378/2007 Coll. (§53) on medicinal products. The relevant Ethics Committee has been contacted and its opinion is part of the supplementary materials. Part of the informed consent to HBOT treatment in our hospital is consent with reverse viewing and analysis of medical charts and the anonymous processing of data from the health documentation, which is used exclusively for scientific and educational purposes.

### Data availability statement

The data that support the findings of this study are available from the first author (MH.) upon reasonable request.

## Results

### Demographic data

In 2019-2023, a total of 21 patients have been treated with hyperbaric oxygen in our unit, of which 11 (52%) males and 10 (48%) females. The mean age was 6±4.6 years (median 4, range 1-15). 20 patients (95%) were hospitalized in the pediatric department during HBOT therapy, while 1 patient was outpatient. Of these 20 hospitalized cases, hospitalization was provided at the City Hospital Ostrava in 19 cases and in one case at the University Hospital Ostrava, when the patient was transported to HBOT by ambulance on a daily basis. Regarding the location of primary workplace, a total of 5 hospitals were involved. The dominant one was the University Hospital Ostrava (9 cases, 43%), followed by the University Hospital Brno and the University Hospital Olomouc with 4 cases (19%).

There were 12 cases of TBI, 8 cases of HIE after CPR (2 cases of TBI followed by CPR and HIE), and 1 case of ischaemic stroke after cardiac surgery procedure. Initial GCS score was 3.3±0.9. Several patients underwent surgery (7 cases, 33%) either in relation with trauma (revision, dura mater surgery, haematoma evacuation, decompression surgery, ventricular drainage, osteosynthesis, resection procedures), or in causal relation with brain damage (extensive cardiac surgery for combined heart disease). Intracranial pressure (ICP) was monitored in 6 patients (29%). Extracorporeal membrane oxygenation (ECMO) and controlled hypothermia were administrated as part of primary treatment in 2 patients. In 2 cases, patients were implanted with a pacemaker/ICD.

The mean time from injury to HBOT was 5.2 ± 3.8 weeks (median 4, range 2-20).

### Course of HBOT, discomfort and adverse effects

Twenty-one patients were treated with HBOT at a pressure 2.0 ATA. A total of 209 exposures were applied. The mean number of exposures was 10± 4.3 (range 2-18, median 10). Nineteen children were premedicated prior to each HBOT exposure, usually with sedatives or benzodiazepines (midazolam, chloral hydrate). Intravenous sedation was administered in 17 (81%) patients during the treatment in hyperbaric chamber (sedatives, e.g. chloralhydrate, benzodiazepines such as midazolam, ketamine, neuroleptics, hypnotics such as propofol, dexmedetomidine). Prevention of barotrauma of middle ear cavities was ensured by the insertion of ventilation tubes in 18 cases (85%) and by paracentesis in 2 cases (10%). Seven children (33%) had a tracheostomy cannula inserted at the time of HBOT. Patients were accompanied during treatment inside the hyperbaric chamber in 18 cases (86%) by pediatric nurses, in 3 cases (14%) by pediatrician and hyperbaric nurse.

Total number of patients with difficulties, disorders and adverse effects during HBOT was 19 (90%). Details of patients related to course of HBOT and adverse effects are listed in table 1. The most common causes of problems were psychological discomfort (e.g. psychomotor restlessness, claustrophobia) in 16 (76%) cases, as well as infectious disorders (4 cases, 19%), dysbarism (3 cases, 14%), neurological and respiratory disorders (both per 2 cases, 10%). 12 patients (57%) have interrupted course of HBOT, most often for psychological discomfort (6 cases, 29%), infectious (seropurulent mediootitis, lower respiratory tract infection, and rhinopharyngitis) disorders in 4 cases (19%), and respiratory disorders in 2 cases (10%). Three patients (14%) ceased HBOT. The cause was severe psychological discomfort and intolerance to treatment in 1 case, acute rhinopharyngitis (1 case) and respiratory problems (1 case) resulting from an inspiratory stridor with pathological respiratory pattern and pathological respiratory mechanics. There was no serious HBOT-related complication or injury.

### Assessment of consciousness and functional and prognostic status

The GCS and GOS was evaluated twice in every patient, at the beginning and end of HBOT treatment. Mean GCS was 10.7±3.7 pre-HBOT, and 12.3±3.4 after post-HBOT, respectively. The difference in results was statistically significant at p=0.004. The mean GOS was 3.3±0.8 pre-HBOT, and 3.9±1.1 after post-HBOT, respectively. The difference in results was statistically significant at p<0.001. The mean and median GCS and GOS pre- and post-HBOT are shown in Table 2.

### Response to HBOT

Three cases were excluded from evaluation of response to HBOT as treatment had been ceased prematurely (cases who experienced discomfort and disorders during HBOT described above). Six cases (33%) were evaluated as large clinically significant response (CSR), seven cases (39%) were evaluated as partial response with minimally important difference (MID). Only 5 cases (28%) were evaluated as non-response. The “overall response rate” as the summation of the CSR and MID revealed 13 cases (72%).

### Response to HBOT related to time from injury

Response to HBOT related to time from injury (18 cases) is presented in table 3. The data show that there is an association among CSR, MID and “no response” results in relation to the duration of injury. There were 6 cases in the category of disease duration up to 4 weeks for the CSR result compared to 0 cases in the category of disease duration more than 5 weeks. Similarly, there were 3 cases in the category of disease duration up to 4 weeks for the MID result compared to 4 cases in the category of disease duration more than 5 weeks. The opposite association is in the category “no response “. The results were statistically significant (Pearson Chi-Square Test, p=0.02).

## Discussion

### Evidence of clinical efficacy of HBOT in brain injury

Over the past decade, there has been a substantial accumulation of evidence regarding the clinical efficacy of HBOT in the field of brain injury. A plethora of experimental studies focusing on severe TBI and HIE have underscored the positive impact of HBOT on apoptosis reduction, cerebral edema mitigation, attenuation of secondary cerebral damage, and enhancement of the blood-brain barrier integrity 16, 25-27.

### Adult population

However, within the domain of clinical medicine, the bulk of scientific evidence available in current literature predominantly pertains to TBI. A Cochrane Review evaluated seven studies involving 571 individuals 28. The findings from two studies indicated that HBOT, compared to control groups, led to a statistically significant decrease in the proportion of individuals with adverse outcomes one month post-treatment (p = 0.001) and also resulted in a significant reduction in mortality (p = 0.003), with a number needed to treat (NNT) parameter of 7. Additionally, two studies demonstrated a reduction in intracranial pressure (p = 0.01), while two other studies highlighted significant improvements in GCS scores among patients treated with HBOT, with an average increase of 2.68 points (p < 0.0001) 28.

However, the question of the use of this treatment and application of hyperoxia in the acute phase of injury, associated with the risk of transport, secondary damage, at the peak of activation of various pathophysiological cascades, is not yet convincingly answered and remains a big question mark. The admission of patients and their treatment is extremely demanding from the medical, organizational, technical and personnel point of view. In contrast, a number of papers have been published on positive results of application of HBOT in the subacute or chronic phase of TBI in the last decade 19-20, 29.

In a systematic review published in 2016, authors reported higher post-treatment GCS scores in the HBOT group compared to the control group, with a combined mean difference of 3.13 (95% CI 2.34-3.92, p <0.001) across eight studies. The authors concluded that patients receiving hyperbaric therapy experienced significant enhancements in both GCS and GOS, along with reduced overall mortality rates. These findings suggest the potential of HBOT as a beneficial component of standard intensive care regimens for traumatic brain injury 30. A Pubmed search in 2018 identified 30 studies (8 clinical and 22 preclinical) that administered HBOT within 30 days of TBI. The results from both preclinical and clinical studies have consistently indicated that HBOT significantly enhances physiological parameters without eliciting adverse effects on brain or lung toxicity, potentially leading to improved clinical outcomes 13.

In another systematic review 31, the authors concluded that HBOT may offer benefits as a relatively safe adjunctive therapy for the acute treatment of moderate to severe TBI. The coherence of clinical trial findings further supported the development of the HOBIT study. HOBIT is proposed as an adaptive clinical investigation aimed at addressing these inquiries and furnishing critical data to inform the design of a definitive Phase III efficacy study 13. The HOBIT trial is designed with two primary objectives: To identify the optimal combination of HBOT treatment parameters likely to showcase enhanced neurological outcomes at the 6-month mark following severe TBI in a subsequent definitive trial. To ascertain whether the chosen HBOT regimen exhibits a probability exceeding 50% of significantly enhancing favorable neurological outcomes at the 6-month mark following severe TBI in a subsequent definitive trial 32. This trial will specifically enroll a targeted subset of patients with severe TBI, totaling 200 subjects over a duration of 3.5 years.

In a recent multicenter, randomized, stratified, controlled prospective clinical trial 158 patients with moderate TBI were divided into four groups: Control group: Received once daily routine rehabilitation training (1/d) without HBOT. Study group A: Received routine rehabilitation training (1/d) with HBOT. Study group B: Received twice daily (2/d) intensified rehabilitation training with HBOT. Study group C: Received twice daily (2/d) intensified rehabilitation training without HBOT 33. These interventions were administered over a period of 3 months. In this study, cognitive ability, activities of daily living (ADL), and exercise capacity were evaluated before and after rehabilitation training using various assessment tools including the Fugl-Meyer Assessment (FMA), Functional Independence Measurement (FIM), Modified Barthel Index (MBI), and Mini-Mental Status Examination (MMSE). Following 1, 2, and 3 months of rehabilitation training, all patients with TBI exhibited significant improvements in FIM, FMA, MBI, and MMSE scores, with particularly notable enhancements observed in patients undergoing twice daily intensified rehabilitation training with HBOT (p < 0.01). The authors concluded that early intensified rehabilitation training combined with HBOT is more advantageous in restoring cognitive, ADL, and physical abilities in patients with TBI 33.

The extensive very recent literature review of clinical recommendations in patients with TBI provides the following summary: Hyperbaric oxygen therapy may be recommended in acute moderate-severe traumatic brain injury patients (Type 2a recommendation, level A evidence). However, further studies are needed to both evaluate outcomes and to determine the optimal treatment protocols for the different types of injuries (Type 1 recommendation, level A evidence). Hyperbaric oxygen therapy should be recommended in chronic traumatic brain injury for a selected group of patients suffering from prolonged post-concussion syndrome who have clear evidence of metabolic dysfunctional brain regions as determined by neuroimaging (Type 2a recommendation, level B-R evidence). Patients should be properly evaluated by standardized cognitive tests and functional brain imaging (Type 1 recommendation, level B-R evidence) 34.

### Pediatric population

The study and control groups (both involving 28 pediatric patients) were compared in terms of duration of hospitalization, GCS, disability reduction, and social behavior. Patients who received HBOT were significantly better than the control group on all the parameters with decreased hospital stay, better GCS and reduction in disability 35.

A total of 15 children with severe TBI who were treated in the pediatric intensive care unit (PICU) were enrolled in this study. Patients with prolonged loss of consciousness after discontinuation of acute treatment in PICU were indicated for HBOT. In patients treated with HBOT, PRISM at admission and GCS after acute treatment were significantly different from patients not treated with HBOT (p < 0.001; p = 0.003, respectively). In other words, patients undergoing HBOT were in a worse state with respect to neurological function. The median time from injury to initiation of HBOT was ± 17.7 days (10-22 days). The average number of completed HBOT sessions was ± 10.9 (3-22). There was no statistical difference in GOS after six months of injury between patients who were treated with HBOT and the untreated group. The authors concluded that HBOT may be beneficial as an adjunctive treatment for children with persistent disturbance of consciousness after acute treatment for severe TBI without significant side effects 36.

Another recent randomized, placebo-controlled, double-blind study investigated the effect of HBOT on children aged 8-15 years experiencing persistent post-concussion syndrome (PPCS) due to mild to moderate TBI events occurring six months to 10 years prior 37. Twenty-five children were randomly assigned to either 60 sessions of HBOT (n = 15) or placebo (n = 10). Following HBOT, significant improvements were observed in cognitive function, including total cognitive score, memory, and executive function, as well as in PPCS symptoms such as emotional and behavioral symptoms. Notably, improvements were also noted in microstructural changes observed in brain MRI for specific brain regions. The study suggests that HBOT can enhance cognitive and behavioral function, alleviate PPCS symptoms, and improve quality of life in pediatric patients with PPCS, even years after the traumatic event 37.

### Safety of HBOT

Treatment with hyperbaric oxygen is generally considered safe treatment procedure, yet there are some complications and side effects. The most prevalent complication of HBOT is barotrauma of the middle ear, with an incidence of approximately 2%. Sinus barotrauma is another reversible complication that commonly occurs in patients with upper respiratory tract infections or allergic rhinitis 38. Reversible myopia, attributed to direct oxygen toxicity to the lens, is also observed in some patients. While the exact cause is unclear, this condition typically resolves within days to weeks after the final treatment session 38. Barotrauma of the lungs, although rare, represents the most severe and life-threatening complication 39. Other pulmonary adverse effects, such as pulmonary edema, chest tightness, and cough, have been seldom reported in association with HBOT 40. Seizures stemming from central nervous system oxygen toxicity are a rare but dramatic consequence of HBOT 41.

A retrospective analysis documented adverse events in 406 out of 2,334 patients (17.4%) who underwent HBOT. The overall incidence rate was calculated to be 721 events per 100,000 sessions (0.72%) 41. Subjective symptoms of barotrauma, such as pain, were reported by 79 individuals (3.4%), while 215 patients (0.36%) exhibited objective signs of middle ear barotrauma upon otoscopic examination. Additionally, 16 patients (0.02%) presented with objective sinus barotrauma. A total of 58 patients (2.5%) discontinued the prescribed HBOT sessions due to side effects, with middle ear barotrauma accounting for the majority (55%) of treatment terminations 41, 42.

Complications in adult patients in intensive care regimen is by no means a rarity. In a European multicentric observational study the rate of adverse events in patients requiring intensive HBOT care was less than 19%, i.e. ten times more often than in patients treated for chronic diseases 43. In addition, in children compared to adult patients can be expected to have high levels of anxiety and fear from a foreign environment, restlessness and non-cooperation e.g. during maneuvers necessary to compensate for pressure changes in middle ear cavities 7.

### Current practice on the field

Treatment of pediatric patients in hyperbaric medicine units even in a very serious medical condition can be realized, but its provision is challenging and requires special measures of operational, technical, organizational and personnel nature 44, 45. Our study concerned pediatric patients with brain injury, who are a regular part of our common operational program for decades. Treatment is fully covered by the public health insurance system of the Czech Republic, and patients or relatives do not pay any co-payment. Brain injury is placed on the official list as optional indication for HBOT in the Czech Republic 7. The situation in this area is on the contrary at such a level that the rejection of a suitable patient in this clinical situation is considered unethical.

This condition similarly appears on the lists of approved indications of major professional societies such as on the list of indications of the 10th ECHM Consensus Conference 2016, as Type 3 - optional indication “Brain injury in highly selected cases” (acute and chronic phase of traumatic brain injury, chronic phase of stroke, post-anoxic encephalopathy) 46.

### Limitations and strengths of the study

We are aware this study has several methodological limitations, which are underlined by the study design which we had to choose. The retrospective case series has descriptive observational study design. There was no control group in this case series and no control for multiple comparisons. We were seeking for the controlled group, however we failed to identify a larger sample. The main limitation is the heterogeneity of the patients inherent in the diagnosis of TBI and HIE (causes of injury, circumstances, symptoms, time from injury to treatment, etc.). Although the operational area is quite large, the catchment area has a population of about 4.5 million, suitable pediatric age patients are so few that it is very difficult to perform smaller homogeneous groups of patients in a real time window. The solution is a multicenter study involving other pediatric departments as well as other hyperbaric centers in the western part of the country (Prague, Kladno, etc.).

The sample size is also limited to the capacity of our hyperbaric unit. On the other hand, all suggested patients were consecutively admitted to our center with some preselection. Although our statistical analyses are performed on a small sample on the before and after data assessment on one sample, the change in absolute numbers suggests a potential of HBOT in the treatment of brain injury, which should be further studied with properly designed RCT. The data were collected by one experienced trained nurse, and data were analyzed by another member of staff, so we tried to limit some of the measurement biases.

### Recommendations for further research and practice

Based on our literature review and our own results, we recommend for future research to design a multicentric randomized controlled study with optimal information size, which includes patients ideally up to four weeks after brain injury. Objective measurement of functional outcomes should be included and patients in both groups should have similar baseline characteristics, including their other treatments.

### Implication for practice

The certainty of the existing evidence on the effectiveness of HBOT in brain injury patients is low, but it shows both a clinical and statistically significant (p<0.05) effect on improvement of the functional neurological state. Guideline developers should consider developing conditional recommendations for the use of HBOT in those patients until the study with proper design and sample size is completed.

## 5. Conclusions

Results of our study demonstrate both clinical and statistically significant patient response to HBOT. Our data also suggest that the earlier HBOT started after diagnosis (up to 4 weeks inclusive), the more pronounced patients' response to HBOT was achieved. The provision of HBOT to pediatric patients is feasible in large regional hyperbaric centers.

## Supplementary Material

Supplementary information.

## Author Contributions

MH- Conceptualization, methodology, formal analysis, software, data curation, writing—original draft preparation. DCh—suggestions, funding acquisition, writing—review and editing. OJ—supervision, project administration, suggestions, writing—review and editing JT—suggestions, writing—review and editing. All authors have read and agreed to the published version of the manuscript.

## Figures and Tables

**Table 1:** Course of hyperbaric oxygen therapy (HBOT), discomfort, adverse effects

	n	%
patients with difficulties during HBOT	19	90
psychomotor restlessness, discomfort, claustrophobia	16	76
infectious disorders	4	19
dysbarism	3	14
neurological disorders	2	10
respiratory disorders	2	10
HBOT discontinuation	12	57
premature cessation	3	14

**Table 2:** Glasgow Coma Scale (GCS) and Glasgow Outcome Scale (GOS) evaluation pre- and post-HBOT (hyperbaric oxygen therapy)

	pre-HBOT			post- HBOT			
	mean±SD	median	range	mean±SD	median	range	Wilcoxon Test
GCS	10.7±3.7	11	3-15	12.3±3.4	14	3-15	p=0.004
GOS	3.3±0.8	3	2-4	3.9±1.1	4	2-5	p<0.001

**Table 3:** Response to HBOT (hyperbaric oxygen therapy) related to time from injury to HBOT (18 cases)

	time from injury to HBOT			
response to HBOT	up to 4 weeks	5 weeks or more	sum	Pearson Chi-Square Test
no response	1	4	5	
Minimally Important Difference -MID	3	4	7	p=0.02
Large Clinically Significant Response - CSR	6	0	6	
sum	10	8	18	

CSR-clinical significant response, MID-minimally important difference
