# Supplementary material for: Hyperbaric Oxygen Therapy in Children with Brain Injury: A Retrospective Case Series
Source: Int J Med Sci. 2025 Jan 1;22(3):473–81. doi: 10.7150/ijms.102884 (PMC11783073; doi:10.7150/ijms.102884)
Supplement: Supplementary file 1 — Supplementary information. [file ijmsv22p0473s1.pdf]

Informovaný souhlas

Léčba kyslíkem za podmínek zvýšeného tlaku – hyperbarická oxygenoterapie

Pacient (štítek)

Jméno a příjmení:

Rodné číslo: **Bydliště:**

Zákonný zástupce

Jméno a příjmení: **Datum narození:**

Vážená paní, vážený pane, vážení rodiče,
v předkládaném formuláři si přečtete informace o léčbě kyslíkem za podmínek zvýšeného tlaku – hyperbarické oxygenoterapii.
Pomohou Vám připravit se na rozhovor s lékařem, který Vás bude informovat o navrhovaném postupu, abyste se mohl(a) rozhodnout a dát souhlas k jeho provedení.

Co je hyperbarická oxygenoterapie:
Hyperbarická oxygenoterapie je léčebná metoda (dále HBO) spočívající v inhalaci 100 % kyslíku za podmínek tlaku vyššího, než je tlak atmosférický. Zvýšeného tlaku lze dosáhnout pouze v uzavřeném zařízení, které se nazývá hyperbarická (tlaková, přetlaková) komora. Tlakové komory jsou zpravidla ocelové tlakové nádoby, jejichž provoz podléhá přísným bezpečnostním opatřením a speciálním technickým normám (např. vícemístné komory normě ČSN EN 14 931). V našem zařízení používáme vícemístnou komoru plněnou upraveným medicinálním vzduchem dle normy ČSN EN 12 021.

Jaký je důvod (indikace) tohoto výkonu:
K léčbě HBO jste byl indikován z toho důvodu, že tato metoda je v kombinaci s běžně užívanou léčbou považována za prospěšnou a vhodnou a může zajistit příznivější průběh Vašeho onemocnění.

Jak to může fungovat:
HBO přináší několik desítek fyziologických efektů a změn nejen v oblasti transportu a metabolismu kyslíku, ale také na kardiovaskulární, respirační, neurologické, mikrocirkulační, metabolicko-biochemické, imunitní, enzymatické a genetické úrovni. Nabídka kyslíku může být až 15násobně vyšší než při dýchání vzduchu za normálních podmínek.
Mnohonásobné zvýšení parciálního tlaku kyslíku v krvi (hyperoxie) přináší celou řadu účinků. Jen příkladem – prodloužení transportní (difuzní) vzdálenosti kyslíku směrem od vlásečnice k buňce, zvýšení dostupnosti kyslíku ve tkáních, přesměrování krevního toku do tkání se sníženou dostupností kyslíku ve tkáních (hypoxie), snížení tkáňového otoku, dále smrtící efekt vůči některým druhům bakterií. Dosažená hyperoxie a vzniklé reaktivní formy kyslíku a dusíku se podílí na modulaci imunitních funkcí, zesílení boje proti infekci a urychleném hojení nehojicích se ran a defektů, obzvláště spojených s bakteriální infekcí. Vše podstatné o HBO najdete na webových stránkách **www.hbova.cz.**

Jaký je režim pacienta před výkonem:
Na našem pracovišti jsou ošetřováni pacienti v celé šíři indikačního spektra, léčíme dospělé pacienty i děti. Pacienti v stabilizovaném celkovém stavu pro chronická onemocnění mohou docházet ambulantně, léčíme však také kriticky nemocné pacienty v emergentním režimu pro akutní onemocnění, kteří jsou k léčbě přivezeni sanitou nebo letecky a jsou hospitalizováni na standardním lůžkovém oddělení, jednotce intenzívní péče či dokonce na resuscitačním oddělení.
Před výkonem samotným nejsou nutná specifická režimová opatření. Je však nutno respektovat omezení a zákazy z důvodů požární bezpečnosti.

Platí přísný zákaz nosit do komory:

- hořlavé látky jako zápalky, zapalovače, kuřácký materiál, těkavé látky – alkohol či dezinfekci a kosmetické přípravky na bázi alkoholu, dále oleje, masti, spreje, rtěnky
- je nutné rovněž odstranit make-up z obličeje a léčebná krytí na defektech a ranách, která obsahují složku na tukové či olejové bázi (Betadine mast, Inadine, Yellonet, Adaptic, Grassolind, Bactigras, Ialugen, Atrauman, Atrauman Ag apod.)
- mobilní telefony, CD, DVD, MP3 přehrávače, iPody, hodinky, baterie, igelitové tašky, noviny, protézy, naslouchadla, diabetická čidla, paruky a příčesky
- v komoře mohou být používány pouze přístroje certifikované pro pobyt v přetlaku
- oblečení a přezůvky ze syntetických materiálů – vhodný materiál je bavlna
- oblečení Vám bude bezplatně poskytnuto
- oblečení, příruční zavazadla je nutné zamknout ve vyhrazeném prostoru
- jídlo – není povoleno – pouze diabetici mají povolenou suchou stravu bez mastnoty (např. suchý rohlík)

Jaký je postup při provádění výkonu:
Pacienti inhalují kyslík skrze těsnicí masku z dýchacího přístroje, jenž se nazývá dýchací automatika. Svým nádechovým úsilím otevírají přívod kyslíku. Dětem lze kyslík podat dětským anesteziologickým systémem. Pacienti v bezvědomí jsou ventilováni plicním ventilátorem přes orotracheální či tracheostomickou kanylu.
Na počátku kompresní fáze pacienti pociťují tlak v uších. Dochází k tomu proto, že prostor v dutině středoušní za bubínkem se smršťuje při vzestupu tlaku, a naopak expanduje při poklesu tlaku. Aby nedošlo k poškození ušního bubínku při těchto tlakových změnách, je nutno vyrovnat tlakový gradient prouděním vzduchu z nosohltanu skrz Eustachovu trubici. K tomu je třeba provádět různé manévry, např. polknutí, otevření úst, pohyb dolní čelistí, výdech při současně uzavřeném nosu a ústech (Valsalvův manévr). Na počátku léčby při zvýšení tlaku dochází k zahřátí vzduchu, naopak ke konci terapie dochází k ochlazení v souvislosti s poklesem tlaku v komoře. Tyto změny jsou způsobeny fyzikálními zákony, jsou tudíž pouze zčásti ovlivnitelné klimatizací.
Léčba probíhá 1x denně po dobu zpravidla 5–30 dnů s ohledem na indikaci a celkový stav. U vybraných stavů může léčba probíhat 2-3 x denně. Průměrná léčebná jednotka v hyperbarické komoře trvá cca 2 hodiny a má tři fáze:

1. *Kompresi*, při které je hyperbarická komora natlakovaná na příslušný léčebný tlak, zpravidla 0,2 MPa, tedy 10 metrů vodního sloupce.
2. *Isopresi*, při které si pacienti střídavě vdechují kyslík a vzduch, trvající 75–80 minut.
3. *Dekompresi*, při které je komora vypuštěna.

Personál komory pacienty poučí, jakým způsobem vyrovnávat tlaky ve středouší a jakým způsobem dýchat kyslík z masky.

Nemocniční 898/20A
728 80 Ostrava – Moravská Ostrava
T 596 191 111 **F** 596 618 781
IČ 00635162 **www.mnof.cz**

Jaké jsou možné komplikace a rizika:

Léčba hyperbarickým kyslíkem je obecně považována za bezpečnou léčebnou proceduru. Díky zvýšenému proudovému odporu v dýchacích cestách je pobyt v přetlaku prokazatelně namáhavější než v prostředí s normálním tlakem vzduchu. Stlačené plyny mají jednak vyšší hustotu, jednak se při jeho dýchání v dýchacích cestách významněji uplatňuje turbulentní proudění. Ke zdravotním rizikům léčby patří soubor tzv. dysbarických poranění. Jedná se o všechna onemocnění v souvislosti s působením tlaku a jeho změnami.

a) Barotraumata jsou mechanická poranění vzniklá tlakovým rozdílem mezi dutinami vyplněnými plynem (nejčastěji vzduchem) a okolím. Mohou postihnout středoušní dutinu, dutiny v obličejové části lebky, zubní dutiny, zažívací trakt a taktéž plíce. Nejčastější formou je však zpravidla barotrauma středouší lehkého stupně a projevuje se silnými bolestmi ucha. Příčinou je zpravidla zánět s otokem sliznice v oblasti nosohltanu. Tato komplikace se vyskytuje asi ve 3 % případů. V případě, že pacient není schopen tlakový gradient vyrovnat ani po aplikaci nosních kapek či léků, je **terapii nutné na několik dnů** odložit a zánět vyléčit. Pokud je léčba HBO absolutně indikovaná a nelze ji odložit, je nutno provést oboustrannou paracentézu – otvor do bubínku otorinolaryngologickým specialistou. Z výše uvedených důvodů je nutno brát zřetel na stavy spojené s implantací různých zařízení či předmětů do organizmu. Roste počet pacientů, kteří mají ve svém těle implantovaný přístroj, zpravidla kardiostimulátor (KS) nebo kardiostimulátor-defibrilátor (ICD), ev. přístroj pro míšní stimulaci u pacientů s chronickými bolestivými stavy, hloubkovou mozkovou stimulací u Parkinsonovy choroby apod. Před zařazením pacienta k léčbě je nutné vždy ověřit, o jaký typ přístroje se jedná a jestli je vhodný a certifikovaný výrobcem pro použití v hyperbarickém prostředí. Rovněž pacientky s implantovanými prsními implantáty by o této skutečnosti měly informovat ošetřujícího lékaře hyperbarického centra.

b) *Reverzibilní myopie* – změny refrakční síly čočky (v průměru o 0,64 dioptrie), reverzibilní do 6–8 týdnů

c) *Toxicita kyslíku* – je známo, že může postihnout všechny tkáně a orgány v závislosti na parciálním tlaku a délce expozice. **Klinicky nejvýznamnější jsou** CNS (centrální nervový systém) forma a plicní forma (chronická). Toxicitu mohou ovlivnit jisté stavy či medikace (kortikoidy, insulin, adrenalin, noradrenalin, inhalace oxidu uhličitého, horečka). Prevencí je dodržení léčebného režimu a stanoveného počtu expozic.

Jaký je režim pacienta po provedení výkonu:

Neexistují žádná speciální režimová opatření u běžného výkonu, který proběhl bez komplikace.

Jaké jsou možné alternativy výkonu:

V současnosti neexistuje léčebná alternativa výkonu.

Ochrana osobních údajů:

Městská nemocnice Ostrava (dále také jen MNO) provádí z důvodu zajištění poskytování kvalitní a bezpečné zdravotní péče a z důvodu průkazného vedení zdravotnické dokumentace kamerový monitoring při expozici v léčebné komoře Centra hyperbarické medicíny.

Při zpracování osobních údajů je v MNO postupováno v souladu s Nařízením Evropského parlamentu a Rady (EU) č. 2016/679, přičemž nezbytné informace o zpracování osobních údajů jsou ve smyslu článku 13 Nařízení pacientovi poskytovány v rámci dokumentu dostupného v prostorách MNO a na internetových stránkách nemocnice, dostupných pod http://www.mnof.cz/o-nemocnici/ochrana -osobnich-udaju/.

Byl(a) jsem lékařem poučen(a) o významu pořízení audiovizuálních záznamů v průběhu mé léčby, které mohou být v případě mého souhlasu použity pro vědecké a studijní účely a mohou být prezentovány na seminářích, na kongresech, publikovány v odborných časopisech. Záznamy budou pořízeny pouze z těch částí mého těla, které přímo souvisí s léčbou. Při prezentaci nebudou zveřejňovány mé osobní údaje (jméno, příjmení), ani další identifikační znaky mé osoby.

S pořizováním takových záznamů souhlasím: ano □ ne □

Dále jsem byl poučen lékařem o významu zpětného nahlížení do zdravotní dokumentace a anonymního zpracování údajů a dat ze zdravotnické dokumentace. Data budou uložena na bezpečném místě a chráněna před neautorizovaným zveřejněním. Anonymizací dat odpadají jakákoliv rizika jejich zneužití. Tento způsob retrospektivního výzkumu je na našem pracovišti standardně a dlouhodobě prováděn. Slouží výhradně pro vědecké a studijní účely, prezentace na seminářích, kongresech, publikování v odborných časopisech, a obecně k šíření osvěty a vědecko-výzkumných informací.
Se zpětným nahlížením do zdravotní dokumentace a anonymním zpracováním dat ze zdravotní dokumentace souhlasím: ano □ ne □

Dále souhlasím, aby osoby získávající odbornou způsobilost k výkonu zdravotnických povolání (studenti lékařských a farmaceutických fakult, zdravotnických škol, akreditovaných kurzů a učitelky odborné praxe) prováděly u mé osoby pod dohledem pověřeného personálu MNO lékařskou a ošetřovatelskou péči: ano □ ne □

Do mé zdravotnické dokumentace mohou nahlížet v rozsahu nezbytně nutném:

- studenti lékařských a farmaceutických fakult, zdravotnických škol, učitelky odborné praxe: ano □ ne □
- pověření auditoři MNO: ano □ ne □
- pověření zaměstnanci MNO za účelem získání potřebných údajů v oblasti sociálně zdravotní a právní, pro potřeby realizace právních úkonů v zastoupení nemocnice: ano □ ne □

Všechny tyto osoby jsou povinny o získaných informacích zachovávat zákonem uloženou mlčenlivost.

Prohlašuji, že mi byl náležitě objasněn důvod, předpokládaný prospěch, způsob provedení, následky, možná rizika a komplikace plánovaného výkonu. Byly mi vysvětleny možné alternativy včetně jejich komplikací a zdravotní důsledky vyplývající z nepodstoupení plánovaného výkonu. Měl(a) jsem možnost zeptat se lékaře na všechno, co mě ve vztahu k plánovanému výkonu zajímá, a obdržel(a) jsem vysvětlení, kterému jsem porozuměl(a). Lékařem jsem byl(a) poučen(a) o možnosti svůj souhlas s navrženým postupem odvolat.
S provedením výše uvedeného výkonu souhlasím.

Datum:

Podpis pacienta nebo zákonného zástupce	Razítko oddělení, jméno, příjmení a podpis ošetřujícího lékaře, který vysvětlující pohovor provedl

Pacient(ka) není schopen(a) podpisu. Svůj souhlas vyjádřil(a):
popište způsob:

Jméno, příjmení a podpis svědka	Razítko oddělení, jméno, příjmení a podpis lékaře
